# Draft Genome Sequence of Lichen-Forming Fungus *Cladonia metacorallifera* Strain KoLRI002260

**DOI:** 10.1128/genomeA.01065-13

**Published:** 2014-02-13

**Authors:** Sook-Young Park, Jaeyoung Choi, Gir-Won Lee, Jung A Kim, Soon-Ok Oh, Min-Hye Jeong, Nan-Hee Yu, Soonok Kim, Yong-Hwan Lee, Jae-Seoun Hur

**Affiliations:** a Korean Lichen Research Institute, Sunchon National University, Suncheon, South Korea; b Department of Agricultural Biotechnology, Fungal Bioinformatics Laboratory, Center for Fungal Genetic Resources, and Center for Fungal Pathogenesis, Seoul National University, Seoul, South Korea; c School of Systems Biomedical Science, Soongsil University, Seoul, South Korea; d Wildlife Genetic Resources Center, National Institute of Biological Resources, Incheon, South Korea

## Abstract

The lichen-forming fungus *Cladonia metacorallifera* strain KoLRI002260 is capable of producing a number of secondary metabolites, including usnic, didymic, and squamatic acids, which have antitumor, antioxidant, and antibiotic activities. The draft genome assembly has a size of 36,682,060 bp, with a G+C content of 44.91%, and consists of 30 scaffolds.

## GENOME ANNOUNCEMENT

*Cladonia* moss-like lichens are distributed worldwide and are one of the most diverse groups of lichen-forming fungi (1). In Scandinavia and Russia, *Cladonia* species are of economic and ecological significance as a primary food source for reindeer and caribou. Moreover, *Cladonia* lichens are known to produce a number of antitumor, antioxidant, and antibiotic compounds (2–4). These compounds, including depsides, depsidones, depsones, and dibenzofurans, are synthesized via the polyketide synthesis pathway (5).

Recently, a polyketide synthase (PKS) gene was characterized at the molecular and structural levels using *Cladonia metacorallifera* (6). The thallus of *C. metacorallifera* contains more than three compounds, such as usnic, didymic, and squamatic acids (7), suggesting that a number of putative PKS genes are present in this fungus. However, the unavailability of the genome sequence has been a major hurdle in identifying the PKS genes. Here, we present the genome sequence of *C. metacorallifera*.

The strain *C. metacorallifera* KoLRI002260 was isolated from apothecia collected at Mt. Seorak (38°06′42.8″N, 128°24′21.8″E), Gangwon-do, South Korea, in 2004. DNA from axenic culture of the fungus was extracted using a DNeasy minikit (Qiagen, Valencia, CA). Draft sequencing was performed by the Illumina HiSeq 2000 system using a whole-genome shotgun strategy (Macrogen, Inc., Seoul, South Korea). The total length of the assembled genome of *C. metacorallifera* strain KoLRI002260 is 36,682,060 bp, with a G+C content of 44.91%, representing 1,023-fold coverage. The genome was assembled into 30 scaffolds (≥1,000 bp) using the SOAP*denovo* assembler (8), GapCloser version 1.12 (for closing the gaps after scaffolding with SOAP*denovo* version 2.04), and SSPACE version 2.0 (9). Subsequent gene prediction analysis using MAKER (10) yielded a total of 11,361 protein-coding genes. Using the previously developed three gene family pipelines (11–13), 320 transcription factor (TF) genes, 92 cytochrome P450 genes, and 2,213 genes encoding secretory proteins were predicted. In addition, 31 putative polyketide synthase genes, containing ketoacyl synthase, acyltransferase, and acyl carrier domains, were predicted by domain search (14).

The investigation of lichen metabolites is a promising field of study for the discovery of novel high-value chemicals. The genome sequence of *C. metacorallifera* strain KoLRI002260 is a valuable resource for identifying the PKS genes and other genes responsible for the biosynthesis of such chemicals. Furthermore, the genome sequence will serve as a platform to facilitate comparative genomics with other lichen-forming fungi, as well as with other species in the phylum *Ascomycota*.

### Nucleotide sequence accession numbers.

The draft genome sequence of *C. metacorallifera* strain KoLRI002260 has been deposited in GenBank under the accession no. AXCT00000000. The version described in this article is the second version, accession no. AXCT02000000. The scaffold sequences were also deposited in GenBank under accession no. KI911109 to KI911138 (30 scaffolds).
